# Investigation of self-regulation skills in children’s picturebooks: a quantitative content analysis

**DOI:** 10.3389/fpsyg.2026.1916149

**Published:** 2026-08-19

**Authors:** Bahar Gümrükçü Bilgici

**Affiliations:** Department of Child Development, Kastamonu Vocational School of Higher Education, Kastamonu University, Kastamonu, Türkiye

**Keywords:** document analysis, early childhood, emotional self-regulation, multiliteracy, picturebooks, self-regulation

## Abstract

**Objective:**

Early childhood is a key time for developing self-regulation, which underpins school readiness and socio-emotional wellbeing. The aim of the study is to provide a quantitative assessment of the representational affordances of children’s picturebooks and their pedagogical potential to support behavioral, emotional, and cognitive self-regulation when mediated by adult-guided reading practice

**Methods:**

A purposive sample of 120 domestic and translated picturebooks published in Türkiye between 2014 and 2025 was evaluated within a quantitative document analysis design. The Picturebook Evaluation Scale provided an objective measure of the capacity of these books along the three subdimensions of self-regulation.

**Results:**

The results reveal that a substantial proportion of the sample (70.8%) contains features associated with general self-regulation. The books in the behavioral domain performed best, with 80.8% explicitly modeling adaptive actions and impulse control. However, 59.2% of the sample demonstrated partially adequate capacity to support emotional self-regulation. Notably, translated picturebooks scored significantly higher in the emotional domain than domestic literature, which often omitted the internal mental states of characters. Furthermore, a significant positive correlation was observed between the structural and aesthetic qualities of a book (e.g., text-image coherence, absence of stereotypes) and its overall potential to support self-regulation.

**Discussion:**

In summary, these results show that high-quality picturebooks contain significant representational affordances that, beyond early literacy, have the pedagogical potential to support the socio-emotional readiness of young children. Adult engagement with children’s literature through structured dialogic reading practices is necessary to allow children’s literature to better reflect the inner emotional lives of characters so that children can reach their full developmental potential.

## 1 Introduction

Different researchers in the literature have defined self-regulation in various ways. According to Schunk and Zimmerman (1997), self-regulation (SR) is a set of acquired, intentional skills involving the processes of controlling, directing, and planning one’s own cognitions, emotions, and behaviors. Moilanen (2007) defines this concept as the flexible activation, monitoring, and adaptation of one’s behaviors, attention, emotions, and cognitive strategies—guided by internal cues, environmental stimuli, and feedback from others—to achieve personal goals. Theorists such as Baumeister and Vohs (2004), Carver and Scheier (1998), however, view SR as the process by which a system uses information about its current state to modify that state to align with a desired goal or standard. Eisenberg et al. (2007), on the other hand, define SR from emotion-focused perspective as processes through which an individual manages the when, how and with what intensity he / she experiences emotions and how he / she expresses them behaviorally.

The structure of SR is multidimensional and is mainly composed of cognitive, emotional and behavioral subdimensions (McClelland et al., 2010). The cognitive dimension is related to working memory and attentional flexibility (McClelland and Cameron, 2011). The emotional dimension (ESR) (emotional self-regulation) refers to the ability to regulate the intensity, quality, and duration of emotional experiences and arousal that occur during the process of goal attainment (Gagne et al., 2021). However, behavioral self-regulation (BSR) refers to the inhibitory control skills to suppress a dominant and reflexive impulse to show a subdominant response appropriate to the situation (Ponitz et al., 2008; McClelland and Cameron, 2011).

The early childhood stage is critical to the rapid development of SR skills, which form the basis of a child’s readiness for school (McClelland and Cameron, 2011). Children with good SR skills find it much easier to adjust to the demands of social contexts and learning environments (Blair and Diamond, 2008; McClelland et al., 2010). The skills children learn early in life are among the best predictors of later success in school (reading and math skills), in relationships with other children, and in social competence. Children who arrive at school without these skills are at high risk for early school dropout, adjustment problems, risky behaviors, and mental health problems.

According to Kopp’s (1982) developmental model, SR follows a gradual transition process from an external control mechanism (regulation by caregivers) to an internal mechanism (SR) (Karreman et al., 2006; McClelland and Cameron, 2011). In early childhood, adult guidance (scaffolding) is crucial for children to acquire these skills. In the classroom and in daily life, free and symbolic (pretend) play—where children set their own rules and take on roles of empathy and leadership—along with structured games, provide the most ideal environments for practicing emotional and behavioral self-regulation (Bodrova and Leong, 2008). Additionally, goal-oriented intervention programs (such as Kids’ Skills or Tools of the Mind) that consciously and repeatedly apply these skills and guide children toward problem-solving and strategy use significantly support the development of self-regulation (Hautakangas et al., 2022).

The development of SR is based on a complex and dynamic interaction between the child’s biological characteristics and environmental experiences (McClelland and Cameron, 2011). Among biological and internal factors, the child’s temperament, reactivity level, and capacity for “effortful control” are paramount (Morrison et al., 2010). The role of the environment and social factors is crucial in the development of SR. Positive parenting styles (warm, responsive and autonomy-supportive parenting, i.e., positive control and responsiveness) have a positive influence on the development of SR, while authoritarian and punitive parenting styles have a negative influence (Karreman et al., 2006; Kim and Kochanska, 2012). At the neurobiological level, environmental stressors such as poverty, neighborhood violence, and domestic chaos interfere with brain stress hormone levels, which impedes the functioning of the prefrontal cortex and impairs SR capacity (Blair and Ursache, 2011; Evans and Rosenbaum, 2008; Wesarg-Menzel et al., 2023).

Unlike ordinary illustrated books where the text and visuals stand alone, picturebooks aimed at children during the early childhood period (ages 0–6) are multimodal literary works where the text, illustrations, and overall design are interdependent, creating a synergistic meaning (Crawford et al., 2024). To ensure that these books best support children’s linguistic, cognitive and social-emotional development, they must contain certain basic features in terms of form, content and visuals:

Design and Formal Features: Children’s picturebooks are typically 32 pages long, which is appropriate for children’s short attention spans (Strasser and Seplocha, 2007). While features such as flaps, moving arms, or touch-sensitive surfaces can increase engagement, research shows that the more interactive features a book has, the more it detracts children’s attention from the core content of the book (e.g., letters or basic information) and that simpler designs are much more effective for information acquisition and learning (Chiong and DeLoache, 2012).

Content and Language Features: The language of these books should be clear, simple, understandable and fully compatible with the child’s cognitive development level (Doğanay Koç, 2024). The narrative flow should be expressed in a short and straightforward style with a limited number of concepts (around 200 words) that do not overload children’s minds (Strasser and Seplocha, 2007). However, the content should be fun while including themes that allow children to relate to their own experiences, build problem-solving skills, or understand the emotions they encounter in their daily lives (Doğanay Koç, 2024; Schoppmann et al., 2023). Another critically important criterion is that the book’s content avoids stereotypes regarding race, gender, culture, or socioeconomic status and presents a culturally authentic, inclusive world (Crawford et al., 2024).

Visual and Illustration Features: Since reading in early childhood largely occurs through visuals, illustrations either share the narrative responsibility with the text or take it on themselves to a large extent (Serafini, 2024). The quality of visuals is critical for children to successfully transfer new information from the book to the real world (learning transfer); using realistic (highly iconic) drawings or photographs rather than overly caricatured or abstract illustrations directly facilitates children’s learning and generalization of concepts (Ganea et al., 2008; Chiong and DeLoache, 2012). Also, the visuals need to be of high quality, with colors and lines chosen carefully to improve children’s sense of beauty and accurately show the mood of the text (Crawford et al., 2024).

A high-quality illustrated children’s book is a holistic art form combining simple, clear design, realistic but attractive art and a flowing text that echoes the child’s world. Children’s books are categorized into various genres based on the balance of visuals and text, such as concept books, wordless picturebooks, informational picturebooks, and narrative picturebooks (Nikolajeva and Scott, 2001). In the academic literature, a very clear distinction is made between ordinary “illustrated books” and “picturebooks,” a unique literary form (Serafini, 2024). In illustrated books the images are used only to decorate, illustrate, or expand the text which has meaning of its own. However, in a real “picturebook,” the words and images are equal partners, with the images able to carry the weight of the narrative on their own, and the combination of these two different modes provides a synergistic meaning (iconotext) that is far more than the sum of its parts (Strasser and Seplocha, 2007; Kucirkova and Tosun, 2024; Serafini, 2024).

Picturebooks create unique learning environments for the language, cognitive, and socio-emotional development of young children (Strasser and Seplocha, 2007; Crawford et al., 2024). Thanks to rich storylines, character development, and examples of conflict resolution, children’s vocabulary and language skills grow rapidly (Strasser and Seplocha, 2007; Kümmerling-Meibauer et al., 2015; Crawford et al., 2024). At the same time, these books help children develop higher-order cognitive and social skills such as observing the world around them, recognizing different cultural realities, building empathy, and thinking critically (Kiefer and Wilson, 2011; Crawford et al., 2024). Early exposure to visual art forms also has a positive effect on children’s aesthetic perception (Crawford et al., 2024; Kucirkova and Tosun, 2024).

Picturebooks offer a safe, indirect guidance environment in the instruction of emotional and behavioral SR strategies that are important for young children (Garner and Parker, 2018). Children learn to model the strategies they see in books about how characters handle intense emotions such as anger, fear, and disappointment, how they control their impulses and solve problems (observational learning) (Garner and Parker, 2018; Fariman and Irawan, 2024). Shared attention (joint attention) between a parent or teacher and a child during a book reading experience helps a child to focus on an object or topic for a longer period of time, thus developing cognitive SR (Kümmerling-Meibauer et al., 2015).

Additionally, during the book-reading process (“Dialogic reading”), asking the child questions about modifying a character’s situation or adjusting their emotional response (response modulation), and using puppets to act out events in the story (“Step Inside” activities) teaches children internal and external emotional self-regulation strategies such as deep breathing and redirecting their attention (Fariman and Irawan, 2024; Xu, 2024). The development of emotional vocabulary facilitates the child’s ability to recognize and express their internal arousal, thereby enabling them to manage their emotional state appropriately (Fariman and Irawan, 2024; Xu, 2024).

### 1.1 Literature review

#### 1.1.1 Experimental studies and impact research

Schoppmann et al. (2023) investigated the development of the ability of “distraction”—an appropriate and flexible emotional self-regulation strategy—among 3-year old children by means of reading picturebooks. The results indicated that in situations of frustrating waiting, characters in literature may serve as a source of this emotional self-regulation strategy for youngsters’ behavior. Wang et al. (2025) demonstrated that structured picturebook activities greatly enhanced emotional self-regulation abilities (awareness, expression, and regulation) and the use of positive techniques (e.g., cognitive restructuring and seeking help) in preschool-aged children. The authors suggest that picturebooks provide a well-designed environment and narrative frame within which young children can view and comprehend their full range of emotional responses. Reading aloud interventions have been found to have a positive effect on children’s SR skills, especially with regard to attention and impulsive control (Piccolo et al., 2022). This study demonstrates that the SR skills children acquire through the book-reading process also mediate (play a mediating role in) language and cognitive development. Xu (2024) found that “dialogic reading” of emotionally rich picturebooks for children directly improves children’s emotional intelligence, as well as their abilities to recognize, express, empathize, and self-regulate emotions.

#### 1.1.2 Studies on emotional socialization and behavioral guidance

Picturebooks are a major tool for children to learn about and socialize their emotions (Garner and Parker, 2018). Research supports that picturebooks, as recommended by social-emotional learning experts, teach children to identify their own and others’ emotions and use this information to regulate their behavior. Fariman and Irawan (2024) explored the emotional self-regulation strategies of child characters in picturebooks. The study highlights the importance of picturebooks to educate children about healthy strategies of emotional regulation such as changing the situation instead of suppressing their feelings.

#### 1.1.3 Theoretical frameworks and literature reviews

Schapira and Grazzani (2025) reviewed the empirical literature on the effects of shared reading on the development of social and emotional competencies, especially SR, of young children in preschool and early childhood settings. Theoretical discussion of how teachers can use picturebooks to support impulse control and SR skills in children 4–6 (Cooper, 2007). The author analyzed classic works such as Maurice Sendak’s “Where the Wild Things Are” and David Shannon’s “No, David!” through the lens of Vygotsky and Erikson’s developmental theories (autonomy, initiative, and SR). Nikolajeva (2013) argues that picturebooks provide children with an “emotional simulation” and a training ground for empathy and theory of mind skills in real life. In this context, she states that books containing the conflicts and emotional states experienced by characters represent the first and most effective step for a child to manage their emotions (self-regulation).

### 1.2 Purpose of the study and hypotheses

This study aims to empirically assess the representational features of children’s picturebooks and their pedagogical potential to support self-regulation, in its behavioral, emotional and cognitive components, in early childhood. Against this background, the study first seeks to describe the competence level of a selected set of picturebooks to support general and subdimensional self-regulation. Furthermore, the research explores the complex interrelations of the books’ origin (domestic or translated), their general aesthetic and structural quality, and their specific selfregulatory potentials.

In line with this general objective, the study seeks to answer the following research questions:

(1) *What are the levels of SR competence in the general and subdimensions of the books?*
(2) *Is there a significant difference in SR scores between the domestic and the translated versions of the examined books?*
(3) *What is the relationship between the general quality scores of the picturebooks and their SR dimensions?*
(4) *Is there a statistically significant correlation between SR subdimensions of picturebooks?*
(5) *Is there a significant difference between the specific formal and content characteristics of the picturebooks (attractiveness of the cover illustration, text-illustration harmony, illustration aesthetics, vibrancy of colors, narrative integrity, and absence of stereotypes) and their SR scores?*

## 2 Materials and methods

### 2.1 Research methodology

This study was conducted using a quantitative content analysis design aimed at systematically examining the qualities of picturebooks that support self-regulation skills (behavioral, emotional, and cognitive) in preschool-aged children (ages 3–6). Unlike traditional qualitative document analysis, quantitative content analysis refers to the objective quantification of written, visual, or auditory communication materials using predefined rules, coding schemes, and validated scales (Neuendorf, 2017). This method is a key research strategy for drawing unbiased, reproducible, and generalizable inferences from texts and their properties by using statistical analysis of the resulting structured data (Riffe et al., 2019).

Within the limits of the study the synergistic meaning created by the textual and visual elements (iconotext) of the 120 selected picturebooks was not interpreted in a purely qualitative way but was transformed into a quantitative framework. The works were scored at the levels of supporting the subdimensions of self-regulation with the “Picture Storybook Evaluation Scale” developed by Deniz and Gönen (2020), which yielded continuous and categorical variables. In this context, the study is based on the empirical framework where the relations and differences between the independent variables (whether the book is a translation or a domestic work, pictorial aesthetics, text-image harmony, etc.) and the dependent variables (self-regulation subdimension scores) are examined through inferential statistical tests (Fraenkel et al., 2012).

### 2.2 Study group

Within the scope of the research, the corpus selection process was systematically designed to ensure methodological transparency and replicability (Neuendorf, 2017). Initially, a broad range of picturebooks published in Türkiye between 2014 and 2025 was screened. These books were obtained on a voluntary basis from families having at least one child. The number of books gathered from each family was not uniform, and no tracking data was kept regarding the exact number of participating families. No financial payment or purchase was involved in the procurement of the picturebooks used in the study. The final sample of 120 picturebooks was selected with a rigorous criterion sampling strategy, which is a purposeful sampling method (Patton, 2015). The inclusion criteria established for the sample were: (a) being a fictional narrative picturebook, (b) being aimed at the preschool education period (3–6 years), (c) being published within the specified 2014–2025 timeframe, and (d) having a multimodal structure in which text and illustrations co-construct the story (Creswell and Poth, 2018).

To maintain comparability and to control for confounding variables, strict exclusion criteria were applied systematically: (a) books explicitly targeting developmental groups outside the preschool period (such as infant-focused 0–2 years books or middle-childhood 7+ years books), (b) text-only books containing no illustrations, (c) wordless picturebooks containing no text (as the evaluation scale requires a multimodal interaction of text and language), (d) purely informational non-fiction books or concept books (e.g., alphabet or number books) designed solely to convey facts rather than narrative play, and (e) books exceeding a page count of 45 pages, which typically represent longer illustrated stories or chapter books aimed at school-aged children with longer attention spans (Strasser and Seplocha, 2007). Additionally, sensory/toy books, poetry collections, fables, and folklore adaptations with no clear narrative plot or character development were excluded. The target age of 3–6 years was verified by cross-checking the official recommendations of the publisher, the cataloging-in-publication (CIP) data, and the developmental guidelines of the Turkish Ministry of National Education. Any books failing to meet these established inclusion and exclusion criteria were systematically excluded. The final corpus of 120 picturebooks was controlled for genre to ensure all selected works fell under fictional narratives. A complete, itemized list of all 120 picturebooks included in this study—detailing their titles, authors, illustrators, publishers, publication years, and publishing origins—is provided in Supplementary Table 1.

As regards the linguistic background of the investigated corpus, 46 picturebooks (38.3%) were domestic books written initially in Turkish and 74 (61.7%) were translated books of different linguistic and cultural origins. The most common original language for the translated books was English (*n* = 57, 47.5% of the total sample, accounting for 77.0% of the translated sub-corpus), followed by German (*n* = 10, 8.3%), French (*n* = 2, 1.7%), Spanish (*n* = 2, 1.7%), Italian (*n* = 1, 0.8%), Slovenian (*n* = 1, 0.8%) and Catalan (*n* = 1, 0.8%). This distribution is representative of a broad spectrum of international children’s literature translated into Turkish, with a dominant representation of Anglophone and Germanic narrative traditions. Table 1 presents the characteristics of the books studied in the group.

**TABLE 1:** Characteristics of picturebooks.

Variables	Characteristics	*n*	%
Writing and Illustration	Turkish	46	38.3
	English	57	47.5
	German	10	8.3
	French	2	1.7
	Spanish	2	1.7
	Italian	1	.8
	Slovenian	1	.8
	Catalan	1	.8
	Translation	74	61.7
Publication year	2016 and earlier	3	2.5
	2017	11	9.1
	2018	18	15
	2019	20	16.7
	2020	20	16.7
	2021	20	16.7
	2022	10	8.3
	2023	13	10.8
	2024 and 2025	5	4.2
Edition of the book	1st ed.	53	44.2
	2nd ed.	14	11.7
	3rd ed.	12	10.0
	4th ed.	11	9.1
	5th ed. or more	30	25.0

### 2.3 Data collection tool and process

In this study, the “Picturebook Evaluation Scale” developed by Deniz and Gönen (2020) was used as the data collection tool to objectively assess the content and illustration characteristics of picturebooks. The scale is presented as Supplementary material. The scale consists of 21 items and a single factor. The scale is designed as a 3-point Likert-type scale with the categories Adequate (3), Partially Adequate (2), and Inadequate (1). Based on the total scores obtained from the scale, books scoring 21–34 points are considered inadequate, those scoring 35–49 points are considered partially adequate, and those scoring 50–63 points are considered adequate. In the process of selecting and categorizing specific items from the Picturebook Evaluation Scale to represent the behavioral, emotional, and cognitive subdimensions of self-regulation, a literature review was first conducted, after which the researcher identified items from the existing scale that belonged to the relevant categories. Then, the identified items were presented to five field experts to ensure the content validity of the study . According to the field experts’ evaluations, items 13, 18 and 19 corresponded to the behavioral dimension of self-regulation, items 6 and 10 corresponded to the emotional dimension and items 9, 11, 14, and 21 corresponded to the cognitive subdimension. The consultation of field experts to verify the extent to which the selected items adequately represent the theoretical structure being measured is a basic validity requirement in the processes of adapting measurement instruments to a specific research objective and the establishment of evaluation criteria (DeVellis, 2017; Lawshe, 1975). The researcher submitted the pairings of the items to the experts to review and verified the internal validity of the study by confirming the items’ consistency with the self-regulation construct based on the feedback received. The CVR (Content Validity Ratio) developed by Lawshe (1975) was calculated for each selected item based on the assessments of the five field experts to quantitatively establish content validity. As presented in Table 2, all selected items achieved a CVR score of either 0.60 or 1.00. Furthermore, the overall Content Validity Index (CVI) for the scale items was calculated as .91, indicating strong expert agreement and robust construct validity for the self-regulation dimensions.

**TABLE 2:** Categorization of picturebook evaluation scale items by SR subdimensions.

Item	SR subdimension	Theoretical justification	CVR value
Item 13: The language and style used in the story are appropriate for the age and developmental level of children.	BSR	Providing developmentally appropriate language and clear external demands helps children understand and comply with expected rules. Behavioral regulation requires the internalization of such environmental standards to flexibly manage overt motor actions and suppress instinctive responses (Calkins, 2007; Kochanska et al., 2001).	0.60
Item 18: The characters in the story are suitable for the child to identify with and take as models.	BSR	Behavioral self-regulation involves observable actions such as persevering in goal-directed behaviors and inhibiting impulsive responses (Deniz and Gönen, 2020). Characters that serve as relatable models allow children to internalize these adaptive actions through observational learning and imitate compliant behaviors in challenging situations (Bandura, 1991; Ponitz et al., 2009)	1.00
Item 19: The story gives importance to ethical and universal values (love, respect, democracy, etc.).	BSR	Emphasizing ethical values provides a framework for socially acceptable behavior and compliance. Behavioral regulation relies heavily on adopting these societal standards to control impulses, focus attention, and act appropriately within social contexts (Calkins, 2007; McClelland et al., 2007).	1.00
Item 6: The characteristics and actions of the characters in the book (body language, emotions, etc.) are accurately reflected in the illustrations.	ESR	Visually representing characters’ emotions and body language helps children recognize and understand internal affective states. This visual scaffolding supports emotion regulation by demonstrating how emotional arousals (such as fear or anger) are experienced, modulated, and behaviorally expressed in challenging situations (Eisenberg et al., 2007; Gagne et al., 2021).	1.00
Item 10: The story is of a quality to meet the psychological/emotional needs of the children.	ESR	Addressing psychological needs creates a secure context for managing internal distress. Emotional self-regulation encompasses the conscious and unconscious strategies used to monitor, evaluate, and suppress or enhance emotional experiences to maintain psychological equilibrium (Calkins et al., 1998; Thompson, 1991).	1.00
Item 9: The subject of the story is appealing/interesting to children.	CSR	An engaging subject captures the child’s interest, which stimulates the voluntary allocation of attention and the use of working memory. These are core executive functions that serve as the foundation for top-down cognitive control and reasoning (Blair and Ursache, 2011; Gagne et al., 2021).	0.60
Item 11: The story nourishes the child’s creative imagination.	CSR	Nourishing imagination fosters cognitive flexibility, allowing children to mentally represent alternative scenarios and develop complex mental strategies. This mental mapping and proactive control are essential components of cognitive self-regulation (Gagne et al., 2021; Miyake et al., 2000).	1.00
Item 14: The style of the story is not preachy and didactic.	CSR	A non-didactic narrative style requires children to utilize metacognitive strategies and deductive reasoning to independently infer meanings and construct knowledge, rather than passively receiving instructions. This active problem-solving heavily exercises their cognitive self-regulatory capacities (Demetriou, 2000; Serafini and Reid, 2022).	1.00
Item 21: The story presents the child with problems they can solve.	CSR	Encountering solvable problems directly engages top-down cognitive abilities, such as planning, directing attention, and organizing thought and action to reach a goal. The conscious application of these executive functions to map out solutions defines cognitive self-regulation (Blair and Ursache, 2011; Miyake et al., 2000).	1.00

During the data collection process, the researcher read each of the 120 books at least three times. No records were taken during the first reading of the books. During subsequent readings, the researcher properly completed the assessment tool and took the necessary notes. The analysis of each book took an average of 40 min. The researcher increased the number of readings and image reviews when deemed necessary.

To ensure the consistency, objectivity, and replicability of the findings, a rigorous inter-coder reliability study was conducted following a pre-established training and coding protocol (Krippendorff, 2018; Miles et al., 2020). First, the independent second coder (a PhD-holding subject-matter expert in early childhood development) underwent a collaborative training session with the primary researcher. During this training session, five picturebooks (separate from the main sample of 120 books) were co-coded and discussed to align the interpretative criteria for each of the 21 items on the Picturebook Evaluation Scale. Following training, a sample of 31 picturebooks (representing 25.8% of the total sample, consisting of 7 domestic and 24 translated works) was randomly selected. Following training, a sample of 31 picturebooks (representing 25.8% of the total sample, consisting of 7 domestic and 24 translated works) was randomly selected. To prevent cognitive bias, the second coder was completely blinded to the publishing origin (domestic vs. translated status) of each of these 31 books. Both coders independently evaluated these 31 books.

Rather than relying on simple correlation coefficients (e.g., 0.87), chance-corrected agreement indices appropriate for ordinal content analysis were calculated using ReCal OIR (Krippendorff, 2018). At the item level, Krippendorff’s alpha (α) was calculated for all 21 items. For the specific self-regulation subdimension items, the alpha values were calculated as follows: 1.000 (Item 13), 0.559 (Item 18), and 0.407 (Item 19) for behavioral self-regulation (BSR); 1.000 (Item 6) and 0.562 (Item 10) for emotional self-regulation (ESR); and 0.627 (Item 9), 0.893 (Item 11), 0.418 (Item 14), and 0.811 (Item 21) for cognitive self-regulation (CSR). For items where the percent agreement was exceptionally high but the alpha fell to zero (e.g., 95.8% agreement, α = 0 for Item 8; 96.8% agreement, α = 0 for Item 12), the result was identified as a classic marginal distribution paradox (or Kappa paradox) caused by low variance and extreme prevalence in the ratings rather than actual coder disagreement (Feinstein and Cicchetti, 1990). At the subscale level, a Two-Way Mixed-Effects Intra-class Correlation Coefficient (ICC) with absolute agreement was utilized. The subscale-level reliability was found to be highly robust: 0.85 for BSR, 0.88 for ESR, 0.89 for CSR, and 0.91 for TSR. Minor coding discrepancies encountered in the independent evaluations were resolved through iterative discussions and consensus building sessions until 100% agreement was achieved on the final dataset.

### 2.4 Analysis of self-regulation subdimensions and scale items

To systematically examine how picturebooks support preschool children’s SR skills, specific items from the “Picturebook Evaluation Scale” developed by Deniz and Gönen (2020) were selected and classified into three basic sub-dimensions: behavioral, emotional, and cognitive self-regulation. Because of the complex and multidimensional nature of SR, which includes volitional regulation of attention, affect, behavior, and executive function (Blair and Raver, 2015; McClelland and Cameron, 2012; Schunk and Zimmerman, 1997), it was important to develop a strong theoretical background for this evaluation. The content validity of this specific categorization was secured by consulting five field experts. Systematic matching of the scale items to the relevant sub-dimensions was carried out based on their expert feedback and established early childhood psychology literature. Table 2 shows selected original scale items, the self-regulation sub-dimensions to which they were assigned, and scientific rationale for each categorization.

To evaluate the construct validity of the measurement model, Confirmatory Factor Analysis (CFA) was conducted using IBM SPSS Amos 20.0 software. Since the scale items were collected on an ordinal 3-point Likert-type scale, the Maximum Likelihood (ML) estimation method was utilized to estimate model parameters. Model adequacy was evaluated using unstandardized factor loadings (*B*), standard errors (*S.E.*), critical ratios (*C.R.*), *p*-values, standardized loadings (β), inter-factor covariances/correlations, and goodness-of-fit indices.

The parameter estimates and factor loading details obtained from the CFA are presented in Table 3, and the overall model fit indices are summarized in Table 4. The standardized factor loadings ranged from 0.019 to 0.724 for the BSR, and from 0.153 to 0.622 for the CSR. Regarding inter-factor relationships, a statistically significant positive covariance was found between the ESR and CSR dimensions (Cov = 0.053, *S*.*E*. = 0.021, *C*.*R*. = 2.515, *p* = 0.012). The correlation between the BSR and CSR was estimated at *r* = 0.233, whereas the covariance between the BSR and ESR dimensions was not statistically significant (*Cov* = 0.001, *S.E.* = 0.004, *C.R.* = 0.172, *p* = 0.863). As presented in Table 4, the model fit indices were evaluated against standard threshold values (Hu and Bentler, 1999; Kline, 2015). The ratio of chi-square to degrees of freedom (χ^2^/*df* = 1.620), GFI (0.936), AGFI (0.880), RMSEA (0.072), and RMR (0.032) indicated acceptable to good model fit, whereas CFI (0.877) and TLI (0.815) demonstrated borderline fit values.

**TABLE 3:** CFA parameter estimates, standard errors, critical ratios, standardized loadings, and inter-factor relationships.

Dimension/item	Unstandardized estimate (B)	S.E.	C.R. (*t*-value)	*P*	Standardized loading (β)
BSR					
s13	1.000*	–	–	–	0.019
s18	197.258	1143.477	0.173	0.863	0.724
s19	197.357	1144.054	0.173	0.863	0.702
ESR					
s10	1.000*	–	–	–	–
s6	-0.304	0.204	-1.495	0.135	–
CSR					
s9	1.000*	–	–	–	0.622
s11	1.669	0.481	3.469	< 0.001	0.572
s14	0.452	0.351	1.287	0.198	0.153
s21	1.406	0.407	3.453	< 0.001	0.550

*Fixed parameter to set scale (S.E. and C.R. are not computed). ESR ↔ CSR: Covariance = 0.053, *S.E.* = 0.021, *C.R.* = 2.515, *p* = 0.012. BSR ↔ CSR: Correlation (*r*) = 0.233. BSR ↔ ESR: Covariance = 0.001, *S.E.* = 0.004, *C.R*. = 0.172, *p* = 0.863.

**TABLE 4:** Model fit indices from confirmatory factor analysis.

Fit index	Model value	Acceptable threshold value*	Result
χ^2^ (CMIN)	38.873	–	–
*df*	24	–	–
χ^2^/*df* (CMIN/DF)	1.620	≤3 (Good); ≤ 5 (Acceptable)	Good Fit
GFI	0.936	≥0.90	Good Fit
AGFI	0.880	≥0.85	Acceptable
CFI	0.877	≥ 0.90	Borderline/Weak
RMSEA	0.072	≤0.05 (Good); ≤ 0.08 (Acceptable)	Acceptable
SRMR (RMR)	0.032	≤0.08	Good Fit
TLI	0.815	≥0.90	Poor

*Threshold values are based on Hu and Bentler (1999), and Kline (2015).

## 3 Results

This section presents data on the 120 picturebooks published for children aged 3–6which were selected from the publications of various publishers and constitute the study group. Deniz and Gönen (2020) noted that, in terms of the quality of picturebooks, books with a total score of 21–34 points are considered inadequate, those with 35–49 points are partially adequate, and those with 50–63 points are adequate. Table 5 presents the adequacy levels of the study group books.

**TABLE 5:** Adequacy levels of the study group’s books.

Writing and illustration	Inadequate		Partially adequate		Adequate	
	*n*	%	*n*	%	*n*	%
Translation	–	–	16	55.2	58	63.7
Domestic	–	–	13	44.8	33	36.3
Total	–	–	29	24.2	91	75.8

In this context, it was determined that 29 (24.2%) of the 120 books included in the study were partially adequate, while 91 (75.8%) were adequate. Based on the calculated range width coefficients, integer boundaries were determined, and the data set was divided into three categories according to these criteria (Table 5).

Using the ranges provided in Table 6, the frequency and percentage distributions of the books in the study group according to the subdimensions of SR are presented in Table 7, which outlines the adequacy scores for the evaluated books across general self-regulation and its specific subdimensions.

**TABLE 6:** Cut-off scores for categories.

Subdimension	Number of items	Inadequate	Partially adequate	Adequate
BSR	3	3–5	6–7	8–9
ESR	2	2–3	4–5	6
CSR	4	4–6	7–9	10–12
Total self-regulation score (TSR)	9	9–14	15–21	22–27

**TABLE 7:** Distribution of books according to SR subdimensions.

Subdimension	Inadequate		Partially adequate		Adequate	
	*n*	%	*n*	%	*n*	%
BSR	5	4.2	18	15.0	97	80.8
ESR	6	5.0	71	59.2	43	35.8
CSR	12	10.0	46	38.3	62	51.7
TSR	2	1.7	33	27.5	85	70.8

The extensive table showed that most of the books are in the “adequate” category (*n* = 85, 70.8%) for total self-regulation scores. While 27.5% of the books (*n* = 33) were found to be “partially adequate,” the proportion of books generally deemed “inadequate” was limited to only 1.7% (*n* = 2).

It was determined that the area in which the books under review were strongest was BSR. In this subdimension, 80.8% of the books (*n* = 97) were coded as “adequate,” while the proportion of books deemed inadequate remained at 4.2% (*n* = 5). It was observed that the books’ performance in ESR skills support differed from that in other dimensions. In this subdimension, more than half of the books were evaluated as “partially adequate” (*n* = 71, 59.2%). The percentage of books that were considered fully “adequate” for emotional support was 35.8% (*n* = 43), which is the lowest “adequate” rate when compared to the behavioral and cognitive dimensions. In the CSR dimension, around half of the books (51.7%, *n* = 62) had “adequate” content and 38.3% (*n* = 46) were found to be “partially adequate.” On the other hand, it was found that the CSR dimension had the highest share of “inadequate” content (10.0%, *n* = 12).

Therefore, while the books generally appear to have sufficient content to support SR skills, imbalances exist across the dimensions. It can be stated that the books frequently feature representations of behavioral self-regulation, but the emotional dimension remains open to development (partially adequate), and relatively more inadequacies were observed in the cognitive dimension.

Before interpreting the relationship between the books’ original language and their self-regulation adequacy levels, chi-square assumptions were checked. In all subdimensions, due to the small number of books categorized as “inadequate,” expected frequencies in some cells fell below the required threshold of 5 (e.g., expected cell counts for the inadequate category were 1.91 for BSR, 2.30 for ESR, 4.60 for CSR, and 0.77 for TSR). To robustly address this violation of assumptions, exact *p*-values calculated via Monte Carlo simulation (based on 10,000 samples) are reported instead of asymptotic *p*-values. Furthermore, Cramer’s *V* was calculated to reduce reliance on *p*-values and to determine the effect sizes (where 0.10 = small, 0.30 = medium, 0.50 = large effect).

Examining Table 8, the original language of the books was not significantly associated with the adequacy levels of behavioral [χ^2^(2) = 1.185, Monte Carlo *p* = 0.580, *V* = 0.10], cognitive [χ^2^(2) = 1.280, Monte Carlo *p* = 0.533, *V* = 0.10], and total SR (χ^2^ = 0.564, Monte Carlo *p* = 0.782, *V* = 0.07). On the other hand, a statistically significant relationship with a moderate effect size was found between the adequacy levels of the ESR subdimension and the original language of the books [χ^2^(2) = 7.536, Monte Carlo *p* = 0.020, *V* = 0.25]. When examining the frequencies, only 21.7% of domestic books were rated as “adequate” in the emotional dimension, whereas this rate in translated books was significantly higher at 44.6%.

**TABLE 8:** Comparison of SR adequacy levels between the domestic and translated picturebooks.

Subdimension	Adequacy level	Domestic (*n*)	Translation (*n*)	Total (*n*)	χ^2^	*df*	Monte Carlo p	Cramer’sV
BSR	Inadequate	3	2	5	1.185	2	0.580	0.10
	Partially adequate	6	12	18				
	Adequate	37	60	97				
ESR	Inadequate	4	2	6	7.536	2	0.020*	0.25
	Partially adequate	32	39	71				
	Adequate	10	33	43				
CSR	Inadequate	3	9	12	1.28	2	0.533	0.10
	Partially adequate	17	29	46				
	Adequate	26	36	62				
TSR	Inadequate	1	1	2	0.564	2	0.782	0.07
	Partially adequate	11	22	33				
	Adequate	34	51	85				

*p < 0.05.

Since the data did not follow a normal distribution, the Mann-Whitney U test was applied to determine whether there was a significant difference in SR scores based on whether the picturebooks were domestic or translated works. To reduce reliance on *p*-values as suggested in the literature, effect sizes (*r*) for the Mann-Whitney U tests were calculated using the formula $r=\left|Z\right|/\sqrt{N}$(where 0.10 is small, 0.30 is medium, and 0.50 is large).

According to Table 9, no statistically significant difference was found between the scores of the BSR (*U* = 1627.00, *p* = 0.642, *r* = 0.04), CSR (*U* = 1537.00, *p* = 0.366, *r* = 0.08) subdimensions, and TSR (*U* = 1690.50, *p* = 0.950, *r* = 0.01). In all these dimensions, the effect sizes were negligible to small. However, the ESR scores showed a statistically significant difference based on the source of the books (domestic/translation) with a moderate effect size (*U* = 1209.50, *p* = 0.004, *r* = 0.27).

**TABLE 9:** Mann-Whitney *U*-test results for total and SR scores of picturebooks regarding the book’s language of composition.

Subdimension	Group	*n*	Mean rank	Sum of ranks	*U*	*Z*	*P*	*r* (Effect size)
BSR	Domestic	46	62.13	2,858.00	1,627.00	-0.465	0.642	0.04
	Translation	74	59.49	4,402.00				
ESR	Domestic	46	49.79	2,290.50	1,209.50	-2.911	0.004**	0.27
	Translation	74	67.16	4,969.50				
CSR	Domestic	46	64.09	2,948.00	1,537.00	-0.905	0.366	0.08
	Translation	74	58.57	4,312.00				
TSR	Domestic	46	60.75	2,794.50	1,690.50	-0.063	0.950	0.01
	Translation	74	60.34	4,465.50				

**p < 0.01.

This finding indicates that, in the translated children’s books examined, elements related to ESR skills (such as characters recognizing, expressing, and managing their emotions) are addressed at a more pronounced or sufficient level compared to domestic children’s books.

Table 10 shows all values as Spearman correlation coefficients (*r_s_*), rounded to two decimal places. To address potential circularity and part-whole overlap, the table presents correlation analyses utilizing both the overall total quality score (including all 21 items) and a recalculated non-circular total quality score (which strictly excludes the nine items used to construct the self-regulation subscales). Regarding the inter-relations among the self-regulation subdimensions, no statistically significant correlation was found between BSR and ESR (*r_s_* = 0.047; *p*> 0.05) or between BSR and CSR (*r_s_* = 0.103; *p*> 0.05). However, a weak but statistically significant positive correlation was observed between emotional and cognitive self-regulation levels (*r_s_* = 0.187; *p*< 0.05), indicating a shared variance between these two domains as represented in the multimodal narrative.

**TABLE 10:** Results of the Spearman correlation analysis between SR subdimensions and total quality scores.

Variables	1	2	3	4	5
1. BSR	–				
2. ESR	0.047	–			
3. CSR	0.103	0.187*	–		
4. TSR	0.473**	0.426**	0.854**	–	
5. Total quality score (other items)	0.022	0.455**	0.237**	0.316**	–
6. Overall total	0.279**	0.536**	0.621**	0.753**	0.844**

*p < 0.05;

**p < 0.01.

When utilizing the non-circular total quality score (excluding the self-regulation items to control for part-whole overlap), no statistically significant relationship was found with the BSR score (*r_s_* = 0.022; *p*> 0.05). In contrast, a statistically significant positive moderate correlation was identified with the ESR score (*r_s_* = 0.455; *p* < 0.01), and a low-to-moderate positive correlation was found with the CSR score (*r_s_* = 0.237; *p* < 0.01). The total self-regulation (TSR) score also exhibited a positive, moderate, and statistically significant correlation with the non-circular quality score (*r_s_* = 0.316; *p* < 0.01).

When examining the overall total quality score (including all 21 items), all self-regulation dimensions demonstrated statistically significant positive correlations: BSR (*r_s_* = 0.279; *p* < 0.01), ESR (*r_s_* = 0.536; *p* < 0.01), CSR (*r_s_* = 0.621; *p* < 0.01), and TSR (*r_s_* = 0.753; *p* < 0.01). These findings indicate that while the inclusion of overlapping items artificially inflates the correlation coefficients, the significant positive associations between book quality and the emotional, cognitive, and total self-regulation capacities remain robust even when part-whole overlap is strictly controlled. However, the association with behavioral self-regulation disappears entirely once this circularity is removed.

### 3.1 Item-by-item analysis of the support provided by picturebooks for SR subdimensions

To systematically identify the specific book characteristics associated with the subdimensions of self-regulation (SR), the items of the Picturebook Evaluation Scale were analyzed with non-parametric statistical procedures [i.e., Kruskal-Wallis H test followed by Mann-Whitney U test with Bonferroni correction (α = 0.0167)]. The item-by-item analysis showed that the visual and aesthetic properties of the picturebooks are essential determinants of their socio-emotional and cognitive support capacities. Specifically, the visual appeal of the book cover (Item 1) was found to be highly significant for the ESR dimension, confirming that an attractive visual entry point enables children to engage with the emotional atmosphere of the narrative. In addition, the structural alliance of text and illustrations (Item 3), the overall aesthetic value of the imagery (Item 4), and the richness of the colors used (Item 7) were found to be statistically significant and positively related to the books’ ESR, CSR, and TSR scores. These findings provide empirical support for the claim that high quality, multimodally coherent visual designs are not simply decoration for a text but essential scaffoldings for children’s metacognitive processing and safe emotional simulation.

Besides visual attributes, the narrative structure and content-related features of the picturebooks were also identified as being strongly related to the BSR dimension. Analysis showed significantly higher BSR scores for clarity and comprehensibility of the main idea of the story (Item 16) and presence of a coherent narrative structure (introduction, body, and conclusion) (Item 17). Notably, the narrative structural coherence also positively influenced the cognitive and total self-regulation capacities of the books, providing support to the hypothesis that logically structured narratives help children to map out problem-solving sequences and to internalize rules. In addition, the lack of stereotyping of language, religion, ethnicity or sex (Item 20) was a strong predictor of adequacy in both BSR and TSR. This suggests that inclusive and non-discriminatory narratives provide a more flexible and richer framework to model adaptive, goal-oriented behaviors and internal impulse control in early childhood.

## 4 Discussion

The present study quantitatively evaluated the potential of 120 picturebooks to develop SR skills (behavioral, emotional, and cognitive) in children, using the scale of Deniz and Gönen (2020). The statistical findings indicate that a substantial majority of the evaluated corpus (70.8%) demonstrates a robust capacity to support general SR. SR in early childhood is defined as the ability of executive functions to consciously control cognitive, affective, and behavioral processes and is recognized as a strong predictor of school readiness (Blair and Raver, 2015; McClelland and Cameron, 2011). The results of the study are in line with the literature and confirm that good-quality picturebooks are not only a means to improve language and literacy skills but also an important “socialization forum” for children to develop their SR mechanisms (Garner and Parker, 2018; Schapira and Grazzani, 2025).

A notable result is that over half of the corpus (59.2%) showed a limited capacity in the subdimension of emotional self-regulation (ESR), with translated literature scoring significantly higher in this domain compared to domestic works. However, this finding must be interpreted with extreme caution and not be taken to mean an inherent superiority of translated children’s literature over domestic literature. Rather, this statistical discrepancy is largely due to selection and publication biases (Riffe et al., 2019). The translated picturebooks in Turkey are not the average children’s literature of their countries of origin but a highly curated and prestigious subset of global literature. Before Turkish publishing houses decide to acquire the translation rights, these works have already been through several thorough international vetting processes, including a successful run in the domestic market, international recognition, the prestige of the publisher, editorial quality and often prestigious awards (e.g., the Caldecott Medal or Kate Greenaway Medal). Conversely, the domestic picturebook sample represents the broader, non-curated spectrum of national publications and as such is bound to be selection-biased.

And cultural differences in emotional socialization and narrative traditions are significant for this distinction (Garner and Parker, 2018). The resulting translated sub-corpus is dominated by Anglophone and Germanic traditions; these are based on Western literary frameworks that highlight individualistic psychological exploration, explicit emotional representation, and “emotional literacy” (Nikolajeva, 2013). Children’s literature in these traditions is often used as a vehicle for the active socialization of complex, internal mental states. The Turkish domestic picturebook narrative tradition, on the other hand, has historically tended to be more action-oriented, socially-oriented and at times moral-didactic. In these domestic stories, the emotional coping tools (e.g., deep breathing, cognitive reframing, or adult help seeking) of characters are often implicit or absent altogether in favor of social harmony and external action. Therefore, the lower ESR scores in domestic books reflect different cultural and narrative emphases in child-rearing and storytelling rather than a structural or pedagogical deficit. Recognizing these distinct cultural socialization strategies is essential for understanding how different literary traditions prepare young readers for self-regulation.

Regarding cognitive self-regulation (CSR), 51.7% of the books provided optimal support. The borderline significant difference between cognitive adequacy and the total quality score and text-picture harmony indicates the decisive role of the multimodal structure in picturebooks on metacognitive skills. In picturebooks, the synergistic structure of words and images (iconotext) requires children to simultaneously process visual and aural inputs (working memory), to deftly redirect their attention (shifting), and to cognitively fill the textual omissions (Serafini, 2024). Collaborative alignment of text and illustration may enhance children’s understanding of cause-and-effect links and the mapping of problem-solving sequences, thus serving as a strong pedagogical tool to support CSR.

In the domain of behavioral self-regulation (BSR), the observation that 80.8% of the books explicitly modeled adaptive behaviors is likely attributable to the concrete and observable characteristics of physical activities and rules in children’s literature. In this dimension, strong positive correlations were found between BSR scores and the criteria of clarity of the main idea, the story’s structural coherence (introduction, development, and conclusion), and the absence of stereotypes. For children to learn to control their internal impulses, follow rules, and persevere in goal-directed actions, the stories they read must be structured within a logically coherent framework (Cooper, 2007). Schoppmann et al. (2023) experimentally demonstrated that children can learn goal-oriented behavioral strategies—such as “distraction”—to cope with frustration by observing characters in picturebooks. On the other hand, the fact that inclusive works free of stereotypes yield significantly higher BSR scores confirms that a flexible narrative—one that is non-discriminatory and non-didactic—offers children far richer, more adaptive, and healthier social behavior models.

The most fundamental finding encompassing all these results is the positive and statistically significant correlation identified between TSR competence and the non-circular total quality score (*r_s_* = 0.316; *p* < 0.01). This statistical evidence, which remains robust even when accounting for the overlap between part and whole, demonstrates that the aesthetic, textual, and formal design of a high-quality picturebook is in agreement with its pedagogical potential. The ability to support self-regulation grows with the overall increase of the book’s artistic and literary quality.

Interestingly, this relationship is primarily driven by the emotional and cognitive dimensions, whereas behavioral self-regulation does not correlate significantly with non-overlapping quality items (*r_s_* = 0.022; *p* > 0.05). This suggests that while basic behavioral rules and model character actions can be presented in books of varying structural quality, the representation of rich emotional and cognitive coping strategies requires high-level multimodal coherence, aesthetic quality, and non-didactic narratives. Indeed, Wang et al. (2025) emphasize that high-quality, structured picturebooks not only develop children’s abilities but also “reshape the ability-strategy link” and guide children toward positive emotional self-regulation strategies rather than negative coping mechanisms.

### 4.1 Implications for practice and future research

Children’s book creators and publishing houses should transition away from traditional, purely didactic narrative frameworks. Instead of presenting behavioral rules through explicit, preachy instructions, authors should design stories that model complex emotional and cognitive self-regulation (SR) strategies (such as cognitive reframing, positive distraction, or seeking adult support). Illustrators and book designers should also treat the visual as an equal partner to the text, and illustrations should reflect the rich internal mental states, facial expressions, and emotional plasticity of characters. The high quality of multimodal design and aesthetic appeal is not a decoration but an essential cognitive scaffolding.

Adults need to understand that children will not learn self-regulatory behaviors through mere passive exposure to picturebooks. Educators and parents should employ structured dialogic reading and shared book reading practices. Adults can actively scaffold children’s metacognitive processing and facilitate the transfer of SR strategies from the fictional narrative to real-world challenges by asking open-ended questions (e.g., “How do you think the character felt when their toy broke?” or “What could they do to calm down their anger?”).

Future research on this document should overcome the limitations of this document analysis by using randomized controlled experimental designs in preschool settings. Integrating picturebooks identified as highly supportive of SR into classroom curricula and tracking longitudinal changes in children’s executive functions would provide invaluable empirical evidence. Additionally, future research could explore how digital and interactive e-picturebooks influence children’s attention allocation and cognitive self-regulation compared to traditional print formats

## 5 Limitations of the study

Although this study empirically and comprehensively demonstrates the potential of picturebooks to support SR skills, there are some limitations that must be considered when interpreting the findings.

First, the study’s sample is limited to 120 picturebooks (both domestic and translated) published in Türkiye between 2014 and 2025. Although these works, selected using a purposive sampling method, reflect certain quality standards, they do not claim to represent all children’s books available in the global market or those published in different periods.

Second, the study adopted a quantitative content analysis design in which qualitative data was quantified. Although the inter-coder reliability metrics were found to be highly robust (with subscale ICCs ranging from 0.85 to 0.91 and ordinal Krippendorff’s alpha values demonstrating overall strong item-level consensus), thereby demonstrating the consistency and objectivity of the evaluations, the data collection process ultimately relies on the researchers’ perceptions and interpretations of multi-modal texts and visuals (iconotext).

A third limitation concerns the validated structure of the measurement instrument used. In the study, the original unidimensional scale was divided into three subdimensions (behavioral, emotional, cognitive) based on expert opinions, and this structure was tested using Confirmatory Factor Analysis (CFA). Although the model’s fit indices—such as CMIN/DF (1.620) and RMSEA (0.072)—provide good and acceptable values that support the structure, the fact that the CFI (0.877) and TLI (0.815) values fall slightly below the 0.90 threshold should be noted as a methodological limitation.

Finally, this study evaluates the “potential” of picturebooks to support SR through document analysis. Since the actual (real-time) effects of the examined books on children’s behavioral, emotional, and cognitive processes do not involve direct observation or experimental applications with child readers, they fall outside the scope of this study.

## 6 Conclusion

This study demonstrates that picturebooks for the 0–6 age group are not merely leisure activities or-writing preparation materials; rather, they contain rich representational affordances that have the potential to support children’s behavioral, emotional, and cognitive SR capacities and form the foundation for their social-emotional and academic readiness. While the majority of the picturebooks examined in this study demonstrated a robust capacity to support general SR, it was determined that Turkish-language works, in particular, lag behind the international (translated) literature in the dimensions of ESR and “reflection of internal mental states,” and that there is room for improvement.

The empirical results obtained demonstrate that the aesthetic appeal of the cover illustration, the harmony between text and illustration, the coherence of the narrative structure, the clarity of the central theme, and the use of an inclusive language free from stereotypes in a children’s book directly, positively, and statistically significantly predict the book’s potential to support SR skills. In light of these strong findings, the following recommendations can be developed for the field:

When designing picturebooks, the reactions of story characters to challenging situations (anger, fear, disappointment) should not be portrayed as “suppressing emotions,” but rather as age-appropriate and positive emotional self-regulation strategies such as “reframing the situation,” “redirecting attention,” or “seeking social support.” Illustrations should not be a simple reproduction of the text. They should be constructed with multi-layered depth that communicates to the reader the inner emotional world and mental map of the character.

Just passively exposing kids does not help them transfer SR strategies that they learn from books to real-life situations. Adults must use structured dialogic reading and shared book reading practices that include questions such as “How might the character have felt in this situation?” and “If you were in their shoes, how would you control your anger?” while reading with the child.

In future studies, integrating Turkish and translated works identified as highly supportive through this scale into preschool classroom settings using randomized, controlled experimental designs, and examining longitudinal changes in children’s cognitive and emotional self-regulation skills, will provide significant contributions to the literature.

## Data Availability

The original contributions presented in the study are included in the article/Supplementary material, further inquiries can be directed to the corresponding author.
