# Supplementary material for: Investigation of self-regulation skills in children’s picturebooks: a quantitative content analysis
Source: Front Psychol. 2026 Aug 19;17:1916149. doi: 10.3389/fpsyg.2026.1916149 (PMC13534015; doi:10.3389/fpsyg.2026.1916149)
Supplement: Supplementary file 1 [file Data_Sheet_1.zip › Supplementary Materials/Picturebook Evaluation Scale.docx]

Supplementary Material

**Picturebook Evaluation Scale**

1. The cover illustration is engaging.

2. More than half of the book’s pages consist of illustrations.

3. The text and illustrations on the same page are harmonious.

4. The illustrations possess aesthetic value.

5. The illustrations are appropriate for the child’s developmental level.

6. The characteristics and actions of the characters in the book (body language, emotions, etc.) are accurately reflected in the illustrations.

7. The colors used in the illustrations are vibrant and eye-catching.

8. The story’s theme is appropriate for the child’s age and developmental level.

9. The story’s theme is engaging for children.

10. The story is designed to meet children’s emotional needs.

11. The story nurtures the child’s creative imagination.

12. The language used in the story is clear, understandable, and grammatically correct.

13. The language and style used in the story are appropriate for the children’s age and developmental level.

14. The story’s style is neither didactic nor instructional.

15. The story is designed to expand the child’s vocabulary.

16. The story’s main idea or theme is clear, concise, and understandable.

17. The story has an introduction, a body, and a conclusion.

18. The characters in the story are suitable for children to identify with and emulate.

19. The story emphasizes ethical and universal values (love, respect, democracy, etc.).

20. The story does not reinforce stereotypes regarding language, religion, ethnicity, gender, etc.

21. The story presents problems that the child can solve.
