# Supplementary material for: Investigation of self-regulation skills in children’s picturebooks: a quantitative content analysis
Source: Front Psychol. 2026 Aug 19;17:1916149. doi: 10.3389/fpsyg.2026.1916149 (PMC13534015; doi:10.3389/fpsyg.2026.1916149)
Supplement: Supplementary file 1 [file Data_Sheet_1.zip › Supplementary Materials/Table S1.docx]

**Table S1**

*Complete List of the 120 Children's Picturebooks Evaluated in the Study*

| **No** | **Book Title** | **Author / Translator** | **Illustrator** | **Publisher** | **Year** | **Pages** | **Origin** |
| --- | --- | --- | --- | --- | --- | --- | --- |
| 1 | *Umudunu Yitirmeyen Sığırcık* | Filiz Özdem | Seçil Çokan | Yapı Kredi Yayınları | 2020 | 18 | Domestic |
| 2 | *Azimli Küçük Kelebek* | Ross Butach / Büşra Oktay | — | Beyaz Balina | 2022 | 40 | Translated |
| 3 | *Küçük Yıldız Tamircisi* | Sam Hay / Nurten Hatırnaz | Xin Li | Beyaz Balina | 2022 | 28 | Translated |
| 4 | *Ama Bu İnek Yumurtluyor* | Andy Cutbill / Nurten Hatırnaz | Russell Ayto | Beyaz Balina | 2019 | 30 | Translated |
| 5 | *Babamın Sandviçi* | Pip Jones / Nurten Hatırnaz | Laura Hughes | Beyaz Balina | 2019 | 30 | Translated |
| 6 | *Arkadaşım Kaptan* | Susanne Schürman / Ayça Sabuncuoğlu | Ralf Butschkow | Türkiye İş Bankası Kültür Yayınları | 2018 | 23 | Translated |
| 7 | *Arkadaşım Dişçi* | Ralf Butschkow / Ayça Sabuncuoğlu | — | Türkiye İş Bankası Kültür Yayınları | 2018 | 23 | Translated |
| 8 | *Arkadaşım Veteriner Hekim* | Ralf Butschkow / E. Gözde Dönmez | — | Türkiye İş Bankası Kültür Yayınları | 2018 | 23 | Translated |
| 9 | *Çağlar Tanımadığı İnsanlarla Bir Yere Gitmez* | Christian Tielmann / Necdet Neydim | Sabine Kraushaar | Türkiye İş Bankası Kültür Yayınları | 2018 | 23 | Translated |
| 10 | *Dijital Ayak İzini Öğreniyorum* | Yeşim Özen | Oğuzhan Seçir | Abaküs | 2017 | 27 | Domestic |
| 11 | *Hayat Dokuyan İplikler Özgür’ün Kitabı* | Nazlı Eda Noyan | Seçil İskit | Çınar | 2020 | 23 | Domestic |
| 12 | *Hayat Dokuyan İplikler Adalet’in Kitabı* | Nazlı Eda Noyan | Seçil İskit | Çınar | 2020 | 23 | Domestic |
| 13 | *Hayat Dokuyan İplikler Değer’in Kitabı* | Nazlı Eda Noyan | Seçil İskit | Çınar | 2020 | 23 | Domestic |
| 14 | *Hayat Dokuyan İplikler Barış’ın Kitabı* | Nazlı Eda Noyan | Seçil İskit | Çınar | 2020 | 23 | Domestic |
| 15 | *Hayat Dokuyan İplikler Erdem’in Kitabı* | Nazlı Eda Noyan | Seçil İskit | Çınar | 2020 | 23 | Domestic |
| 16 | *Eyvah Mutfakta Fil Var!* | Smriti Halls / Nurten Hatırnaz | Ella Okstad | Beyaz Balina | 2020 | 26 | Translated |
| 17 | *Ben Sandalye Değilim!* | Ross Burach / Nurten Hatırnaz | — | Beyaz Balina | 2018 | 37 | Translated |
| 18 | *Annemin Bavulu* | Pip Jones / Nurten Hatırnaz | Laura Hughes | Beyaz Balina | 2019 | 29 | Translated |
| 19 | *Anne Boz* | Ryan T. Higgins / Nurten Hatırnaz | — | Beyaz Balina | 2018 | 38 | Translated |
| 20 | *Ayılar Kitap Okumaz* | Mark Barry / Nurten Hatırnaz | Katy Halford | Beyaz Balina | 2023 | 30 | Translated |
| 21 | *Dinozorlar Örgü Örmez* | Kes Gray / Nurten Hatırnaz | Nick East | Beyaz Balina | 2023 | 29 | Translated |
| 22 | *Çocuk Olmaya Hakkım Var* | Rue Du Monde / Füsun Önen | Aurelia Fronty | Yapı Kredi Yayınları | 2020 | 41 | Translated |
| 23 | *Acıkmadım ki!* | Rebecca Cobb / Sevgi Atlıhan | Rebecca Cobb | Türkiye İş Bankası Kültür Yayınları | 2021 | 32 | Translated |
| 24 | *En Güzel Tüyünü Kaybeden Kuş* | Yurdanur Ay Paşa | Sezen Aksu Taşyürek | abm | 2023 | 32 | Domestic |
| 25 | *Dişim Gıdıklanıyor* | Nazlı Karaca Kurt | Hilal Özdemir | Perseus | 2021 | 27 | Domestic |
| 26 | *Buz Ördeği* | Alex Nogués / Cansu Akkoyun | Ina Hristova | Türkiye İş Bankası Kültür Yayınları | 2022 | 27 | Translated |
| 27 | *Büyükannem De Kuşları Sever* | Andrew Larsen / İlknur Özdemir | Dorothy Leung | Sia Çocuk | 2023 | 34 | Translated |
| 28 | *Porsuk Babasını Çok Seviyor* | Constanze von Kitzing / Zeynep Alpaslan | Constanze von Kitzing | Türkiye İş Bankası Kültür Yayınları | 2025 | 17 | Translated |
| 29 | *Tavşancan ile Faresu Uyku Vakti Kurbağası* | Axel Scheffler / Berrak İdiman | Axel Scheffler | hep kitap | 2019 | 24 | Translated |
| 30 | *Gergedanlar Krep Yemez* | Anna Kemp / Gülbin Baltacıoğlu | Sara Ogilvie | Pearson | 2019 | 30 | Translated |
| 31 | *Kakaloji Bir Kakanın Bilimsel Yolculuğu* | Saniye Bencik Kangal - Ceren Koçak - Merve Solak Arabacı | Berk Öztürk | Turta Kitap | 2021 | 29 | Domestic |
| 32 | *Çekirdek ile Yumurta* | Alex Latimer / Emre Ülgen Dal | David Litchfield | domingo | 2021 | 28 | Translated |
| 33 | *Sihirli Aynalar* | Mark Baker - Neville Astley / Derya İmer | — | Doğan Çocuk | 2023 | 23 | Translated |
| 34 | *Tütü Düştü* | Corey R. Tabor / Emre Ülgen Dal | — | domingo | 2023 | 36 | Translated |
| 35 | *Pazar Alışverişi* | Dr. Nazife Burcu Takıl | Erişcan Türk | Drk çocuk yayınları | 2021 | 16 | Domestic |
| 36 | *Kaybolan Renkler* | Aleix Cabrera / Adem Uludağ | Rosa M. Curto | Tübitak Popüler Bilim Kitapları | 2017 | 37 | Translated |
| 37 | *Evet Yapabilirim!* | Kendra J. Barrett - Jacqueline B. Toner - Claire A.B. Freeland / Gözde Döneli | Violet Lemay | Sabri Ülker Vakfı Yayınları | 2021 | 27 | Translated |
| 38 | *Çise Bir Su Damlasının Hayat Döngüsü* | Gürcan Demirel - Can Batın Demirel | Aslıhan Olcay | Peta Kitap | 2023 | 28 | Domestic |
| 39 | *Kulaktan Kulağa* | Filiz Özdem | Seçil Çokan | Yapı Kredi Yayınları | 2020 | 29 | Domestic |
| 40 | *Baykuş ile Çobanyıldızı* | Filiz Özdem | Emine Bora | Yapı Kredi Yayınları | 2019 | 32 | Domestic |
| 41 | *Işıkları Dolaşan Çocuk* | Aytül Akal | Serap Deliorman | Uçanbalık | 2016 | 16 | Domestic |
| 42 | *Çok Hayal Kuran Çocuk* | Şermin Yaşar | Andere Andrea Petrlik / Shutterstock.com | Elma çocuk | 2019 | 32 | Domestic |
| 43 | *Cebimdeki Mandalina Ağaçları* | Şermin Yaşar | Meryem Tanrıkulu | Doğan Egmont | 2021 | 30 | Domestic |
| 44 | *Cesaret Sandığı* | Şermin Çarkacı | Oya Diker | Elma çocuk | 2017 | 27 | Domestic |
| 45 | *Beni Ne Renk Sevdin Anne?* | Gülşen Arslan Akca | Gülşen Arslan Akca | Timaş Çocuk | 2019 | 32 | Domestic |
| 46 | *Kırılan Kalpler Nereye Gider?* | Gülşen Arslan Akca | Gülşen Arslan Akca | Timaş Çocuk | 2021 | 32 | Domestic |
| 47 | *Teknoloji Bağımlısı Babam* | Dilek Cesur | Elis Özer Avcı | Enda Yayıncılık | 2022 | 23 | Domestic |
| 48 | *Hoşça Kal Okul* | Tonya Lippert / Gözde Döneli | Tracy Bishop | Sabri Ülker Vakfı Yayınları | 2021 | 27 | Translated |
| 49 | *İki Dilek* | Helena Kraljic / Duygucan Yenidünya | Tina Dobrajc | Eksik Parça | 2020 | 29 | Translated |
| 50 | *İki Arkadaş* | Helena Kraljic / Duygucan Yenidünya | Tina Dobrajc | Eksik Parça | 2020 | 29 | Translated |
| 51 | *Söz Vermiştin Ama Anne* | Aytül Akal | Huban Korman | hep kitap | 2018 | 36 | Domestic |
| 52 | *Babamın Battaniyesi* | Sara Şahinkanat | Ayşe İnan | Yapı Kredi Yayınları | 2019 | 37 | Domestic |
| 53 | *Üç Kedi Bir Dilek* | Sara Şahinkanat | Ayşe İnan | Yapı Kredi Yayınları | 2020 | 26 | Domestic |
| 54 | *T-Reks Restoranı* | Aytuna Dirican | Fatemeh Nazari | Düşyeri Kitap | 2021 | 23 | Domestic |
| 55 | *Karlı Bir Gün* | Anna Milbourne / Şeyma Bayrak | Elena Temporin | Tübitak Popüler Bilim Kitapları | 2019 | 20 | Translated |
| 56 | *Sana Kek Yaptım* | Doç. Dr. Saniye Bencik Kangal | Ayşın Delibaş Eroğlu | Turta Kitap | 2023 | 44 | Domestic |
| 57 | *Yemeyeceğim İşte!* | Alberto Pellai - Barbara Tamborini / Feza Özemre | Elisa Paganelli | Turkuvaz | 2021 | 20 | Translated |
| 58 | *Bulut’un Beslenme Macerası* | Uzm. Dyt. Oya Neva Demirkol | Samed Ergün | Şapka ve Fil | 2019 | 21 | Domestic |
| 59 | *Burundaki Parmak* | Paula Merlan / Zeynep Şen | Gomez | Doğan Çocuk | 2022 | 34 | Translated |
| 60 | *Babam Yanımdayken* | Soosh / Cemre Ömürsuyu Seyis | Soosh | Sev | 2018 | 32 | Translated |
| 61 | *Atatürk, Karıncalar ve Çocuk* | Süleyman Bulut | Nazlı Şahin | Kelime Yayınları | 2023 | 31 | Domestic |
| 62 | *Mamut Yıkama Rehberi* | Michelle Robinson / Melike Hendek | Kate Hindley | Pearson | 2018 | 23 | Translated |
| 63 | *Arkadaşım Pilot* | Ralf Butschkow / Ayça Sabuncuoğlu | — | Türkiye İş Bankası Kültür Yayınları | 2019 | 23 | Translated |
| 64 | *Uçmak İsteyen Köpek* | Kathy Stinson / Meltem Aydın | Brandon James Scott | Türkiye İş Bankası Kültür Yayınları | 2022 | 30 | Translated |
| 65 | *Ya Söz Dinlemezsem?* | Maureen Fergus / Meltem Aydın | Qin Leng | Türkiye İş Bankası Kültür Yayınları | 2020 | 29 | Translated |
| 66 | *Yemek Seçen Kirpi* | Andrea Reitmeyer / Duygu Bolut | Andrea Reitmeyer | Türkiye İş Bankası Kültür Yayınları | 2025 | 24 | Translated |
| 67 | *Büyüyünce Ne Olacağım?* | Frances Stickley / Meltem Aydın | Lucy Fleming | Türkiye İş Bankası Kültür Yayınları | 2024 | 29 | Translated |
| 68 | *Neyin Var Porsuk?* | Constanze Von Kitzing / Leyla Çınar | Constanze Von Kitzing | Türkiye İş Bankası Kültür Yayınları | 2022 | 16 | Translated |
| 69 | *Yavru Timsah Çipiti* | Catherine Rayner / Ali Berktay | Catherine Rayner | Türkiye İş Bankası Kültür Yayınları | 2020 | 31 | Translated |
| 70 | *Yaramaz Can* | Katie Haworth / Ali Berktay | Laura Hughes | Türkiye İş Bankası Kültür Yayınları | 2018 | 26 | Translated |
| 71 | *Leblebi Ve Başındaki Dert* | Tor Freeman / Ali Berktay | Tor Freeman | Türkiye İş Bankası Kültür Yayınları | 2020 | 30 | Translated |
| 72 | *Minik Dinozor-Uçan Dinozor* | Ercan Dinçer | Ercan Dinçer | 5 Renk Yayınevi | 2019 | 16 | Domestic |
| 73 | *Minik Dinozor-Yumurtalar Nerede?* | Ercan Dinçer | Ercan Dinçer | 5 Renk Yayınevi | 2019 | 16 | Domestic |
| 74 | *Cem ile Ceren-Yürüyen Çöp* | Ercan Dinçer | Ercan Dinçer | 5 Renk Yayınevi | 2019 | 16 | Domestic |
| 75 | *Cem ile Ceren-Anne Fare* | Ercan Dinçer | Ercan Dinçer | 5 Renk Yayınevi | 2019 | 16 | Domestic |
| 76 | *Sınıfım Nerede?* | Birsen Ekim Özen | Esin Şahin | Timaş Çocuk | 2021 | 16 | Domestic |
| 77 | *Meyveleri Kim Yemiş?* | Feridun Oral | Feridun Oral | Yapı Kredi Yayınları | 2023 | 32 | Domestic |
| 78 | *Cesur Yürek Hermit Evim Olmadan Asla* | Asiye Yıldırım | Hüseyin Sönmezay | Timaş Çocuk | 2020 | 31 | Domestic |
| 79 | *Şu Yaramaz Tavşanlar* | Ciara Flood / Ali Berktay | — | Türkiye İş Bankası Kültür Yayınları | 2018 | 33 | Translated |
| 80 | *Bu Ayı Çocuk Bakamıyor* | Ruth Quayle - Alison Friend / Gökçe Yavaş | Alison Friend | Altın Kitaplar | 2020 | 29 | Translated |
| 81 | *Ayıcıklar Piknikte* | Georgie Birkett | Georgie Birkett | Mikado Çocuk | 2018 | 18 | Translated |
| 82 | *Roko Püfleyen Küçük Dinozor* | David Bedford / Melike Hendek | Mandy Stanley | Pearson | 2019 | 29 | Translated |
| 83 | *Benim Babam Hayattaki En Maceracı Arkadaşım!* | Kollektif | — | 0-6 Yaş Yayınları | 2022 | 24 | Domestic |
| 84 | *Süper Patates-2 Sebzelerin Gücü Adına!* | Sue Hendra - Paul Linnet / Melike Hendek | Sue Hendra - Paul Linnet | Pearson | 2018 | 24 | Translated |
| 85 | *Güvercin Kakası* | Elizabeth Baguley / Burcu Ural Kopan | Mark Chambers | Marsık Kitap | 2021 | 24 | Translated |
| 86 | *Kulübünüze Üye Olabilir Miyim?* | John Kelly / Murat Murat | Steph Laberis | Ketebe | 2023 | 27 | Translated |
| 87 | *Sincap Kardeş (Yardımlaşma)* | Aziz Sivaslıoğlu | İlknur Ercan | Beyaz Pusula Yayıncılık | 2014 | 16 | Domestic |
| 88 | *Cem ile Ceren Rüzgar Neler Yapar?* | Ercan Dinçer | Ercan Dinçer | 5 Renk Yayınevi | 2019 | 16 | Domestic |
| 89 | *Teo’nun Kaka Kitabı* | Yağmur Artukmaç, Psikolog Pınar Çakır | Nurbanu Asena | Bilgi | 2018 | 19 | Domestic |
| 90 | *Baba ile Bir Gün* | Ruby Brown / N. Beyza Elgin | Tina Macnaughton | 1001 Çiçek Kitaplar | 2020 | 22 | Translated |
| 91 | *Kırmızı Sarı Mavi* | Lysa Mullady / Gözde Döneli | Laurent Simon | Sabri Ülker Vakfı Yayınları | 2021 | 27 | Translated |
| 92 | *Tuvalette Kim Var?* | Jeanne Willis / Tülay Yıldız | Adrian Reynolds | Mikado Çocuk | 2018 | 22 | Translated |
| 93 | *Oyun Zamanı* | Amy Jones / Ece Ali | Cheryl Davies | İndigo Çocuk | 2015 | 16 | Translated |
| 94 | *Neden Uyurum?* | Emilie Dufresne / Ezgi Faturacı | — | Sabri Ülker Vakfı Yayınları | 2022 | 23 | Translated |
| 95 | *Elif Korona Zamanlarında Cesaret Veriyor* | Liane Schneider / Aylin Gergin | Janina Görrissen | Türkiye İş Bankası Kültür Yayınları | 2021 | 21 | Translated |
| 96 | *Petek Böceksever Arıları Kurtardığında* | Catherine Jacob / Sima Özkan | Lucy Fleming | Fom Kitap | 2022 | 26 | Translated |
| 97 | *Kurumuş Ağaçlar* | Alper Tüydeş | Berk Öztürk | Karavan Çocuk | 2021 | 37 | Domestic |
| 98 | *Aranıyor! Hayvanlar Aleminin Suçluları* | Heather Tekavec / Şafak Kılıç | Susan Batori | İthaki Çocuk | 2021 | 30 | Translated |
| 99 | *Çiçek Dilinde* | Sara Levine / Şermin Korkusuz Aslan | Masha D’Yans | Tübitak Popüler Bilim Kitapları | 2023 | 29 | Translated |
| 100 | *Komik Hayvan İsimleri Bakanlığı* | Kes Gray / Canan Bolel | Nikki Dyson | Domingo | 2021 | 24 | Translated |
| 101 | *Mürafa Zürafa Uyanmış Mı? Uyanmamış Mı?* | Eda Albayrak | Funda Akman Aydemir | abm Çocuk ve İlk Gençlik Yayınları | 2019 | 20 | Domestic |
| 102 | *Küçük Deniz Kaplumbağası* | Becky Davies / Şermin Korkusuz Aslan | Jennie Poh | Tübitak Popüler Bilim Kitapları | 2021 | 24 | Translated |
| 103 | *Söz Veriyorum* | Lebron James / Zeynep Dilek Demirel | Nina Mata | Butik | 2020 | 28 | Translated |
| 104 | *Komik Yer İsimleri Oteli* | John Hay - Garry Parsons / Emre Ülgen Dal | Garry Parsons | Domingo | 2025 | 24 | Translated |
| 105 | *Elif Diş Doktoruna Gidiyor* | Liane Schneider / Necdet Neydim | Eva Wenzel-Bürger | Türkiye İş Bankası Kültür Yayınları | 2020 | 23 | Translated |
| 106 | *Küçük Bir Mucize* | Bilge Nur Pembegül | Berk Öztürk | Elma Çocuk | 2024 | 29 | Domestic |
| 107 | *Hokus Pokus Diplodokus Dünyanın İlk Sihirbazı* | Steve Howson / Ayça G. Derin | Kate Daubney | İndigo Çocuk | 2021 | 30 | Translated |
| 108 | *Güvercinlerle Matematik* | Asia Citro / Ali Berktay | Richard Watson | Türkiye İş Bankası Kültür Yayınları | 2023 | 44 | Translated |
| 109 | *Mustafa Kemal Atatürk ve Çocuk* | Yılmaz Özdil | Nalan Alaca | Kırmızı Kedi Çocuk | 2018 | 32 | Domestic |
| 110 | *Mustafa Kemal Atatürk ve Kitap* | Yılmaz Özdil | Nalan Alaca | Kırmızı Kedi Çocuk | 2018 | 32 | Domestic |
| 111 | *Mustafa Kemal Atatürk ve Hayvan Sevgisi* | Yılmaz Özdil | Nalan Alaca | Kırmızı Kedi Çocuk | 2018 | 32 | Domestic |
| 112 | *Küçük Bayan Günışığı* | Roger Hargreaves / Özlem Tutar Sarı | Onur Erbay | Doğan Egmont | 2017 | 31 | Translated |
| 113 | *Küçük Bayan Yardımsever* | Roger Hargreaves / Orhan Cem Çetin | Onur Erbay | Doğan Egmont | 2017 | 31 | Translated |
| 114 | *Bay Hapşırık* | Roger Hargreaves / Özlem Tutar Sarı | Onur Erbay | Doğan Egmont | 2017 | 35 | Translated |
| 115 | *Bay Gıdık* | Roger Hargreaves / Orhan Cem Çetin | Onur Erbay | Doğan Egmont | 2017 | 35 | Translated |
| 116 | *Bay Küçük* | Roger Hargreaves / Özlem Tutar Sarı | Şenol Alanbay | Doğan Egmont | 2017 | 35 | Translated |
| 117 | *Utangaç Papağan Huli* | Göktuğ Canbaba | Biğkem Karavus | Doğan Egmont | 2019 | 12 | Domestic |
| 118 | *Yaramaz Sırma ile Ayıcıklar* | Heather Amery / Nevin Avan Özdemir | Stephen Cartwright | Türkiye İş Bankası Kültür Yayınları | 2017 | 15 | Translated |
| 119 | *Uyuyan Güzel* | Heather Amery / Nevin Avan Özdemir | Stephen Cartwright | Türkiye İş Bankası Kültür Yayınları | 2017 | 15 | Translated |
| 120 | *Cesur Fırfır* | Angela McAllister / Sevgi Atlıhan | Tiphanie Beeke | Türkiye İş Bankası Kültür Yayınları | 2017 | 23 | Translated |

*Note.* N = 120. This table presents the complete, itemized dataset used for the quantitative content analysis of children's picturebooks in early childhood education. Origin categories indicate whether the book is a domestic Turkish work or a translated work. All entries are compiled from the primary study database.
